# Phospholipid Encapsulation of an Anti-Fibrotic Endopeptide to Enhance Cellular Uptake and Myocardial Retention

**DOI:** 10.3390/cells12121589

**Published:** 2023-06-08

**Authors:** Swati D. Sonkawade, Shirley Xu, Minhyung Kim, Sarmila Nepali, Victoire-Grace Karambizi, Sandra Sexton, Steven G. Turowski, Kunpeng Li, Joseph A. Spernyak, Jonathan F. Lovell, Anthony George, Sujit Suwal, Umesh C. Sharma, Saraswati Pokharel

**Affiliations:** 1 Department of Medicine, Division of Cardiology, Jacobs School of Medicine and Biomedical Sciences, University at Buffalo, Buffalo, NY 14260, USA; swatidha@buffalo.edu (S.D.S.);; 2 Laboratory Medicine, Department of Pathology, Roswell Park Comprehensive Cancer Center, Buffalo, NY 14263, USA; 3 Department of Surgical Oncology, Roswell Park Comprehensive Cancer Center, Buffalo, NY 14203, USA; 4 Laboratory Animal Shared Resource, Roswell Park Comprehensive Cancer Center, Buffalo, NY 14203, USA; 5 Translational Imaging Shared Resources, Roswell Park Comprehensive Cancer Center, Buffalo, NY 14203, USA; 6 Department of Physiology and Biophysics, Case Western Reserve School of Medicine, Cleveland, OH 44106, USA; 7 Department of Biomedical Engineering, Jacobs School of Medicine and Biomedical Sciences, University at Buffalo, Buffalo, NY 14260, USA; 8 Department of Biostatistics and Bioinformatics, Roswell Park Comprehensive Cancer Center, Buffalo, NY 14203, USA; 9 Department of Chemistry, Buffalo State University, Buffalo, NY 14222, USA

**Keywords:** liposome, RAW 264.7 macrophages, C57BL/6j, cardiac fibroblasts, myocardial infarction, bioavailability, *N*-acetyl-ser-asp-lys-pro (Ac-SDKP)

## Abstract

Background: Cardioprotective effects of *N*-acetyl-ser-asp-lys-pro (Ac-SDKP) have been reported in preclinical models of myocardial remodeling. However, the rapid degradation of this endogenous peptide in vivo limits its clinical use. Method: To prolong its bioavailability, Ac-SDKP was encapsulated by phosphocholine lipid bilayers (liposomes) similar to mammalian cell membranes. The physical properties of the liposome structures were assessed by dynamic light scattering and scanning electron microscopy. The uptake of Ac-SDKP by RAW 264.7 macrophages and human and murine primary cardiac fibroblasts was confirmed by fluorescence microscopy and flow cytometry. Spectrum computerized tomography and competitive enzyme-linked immunoassays were performed to measure the ex vivo cardiac biodistribution of Ac-SDKP. The biological effects of this novel synthetic compound were examined in cultured macrophages and cardiac fibroblasts and in a murine model of acute myocardial infarction induced by permanent coronary artery ligation. Results: A liposome formulation resulted in the greater uptake of Ac-SDKP than the naked peptide by cultured RAW 264.7 macrophages and cardiac fibroblasts. Liposome-delivered Ac-SDKP decreased fibroinflammatory genes in cultured cardiac fibroblasts co-treated with TGF-β1 and macrophages stimulated with LPS. Serial tissue and serum immunoassays showed the high bioavailability of Ac-SDKP in mouse myocardium and in circulation. Liposome-delivered Ac-SDKP improved cardiac function and reduced myocardial fibroinflammatory responses in mice with acute myocardial infarction. Conclusion: Encapsulation of Ac-SDKP in a cell membrane-like phospholipid bilayer enhances its plasma and tissue bioavailability and offers cardioprotection against ischemic myocardial injury. Future clinical trials can use this novel approach to test small protective endogenous peptides in myocardial remodeling.

## 1. Introduction

Myocardial inflammation and fibrosis are major pathological hallmarks of heart failure. Myocardial inflammation increases the propensity for interstitial collagen deposition, which leads to ventricular stiffness and impaired cardiac function [1,2]. Despite multiple studies identifying macrophage activity and fibrosis as the culprit mechanism of heart failure [3,4,5,6], effective pharmacological agents targeting the fibroinflammatory pathways are not available. There are some studies that have tested the effects of PPAR-gamma agonists in preclinical models of obesity-induced cardiac fibrosis [7], but there are no effective therapeutic agents targeting cardiac inflammation. Some cytotoxic agents were examined but they non-selectively deplete inflammatory cells, which are detrimental to cardiac function [8]. The only therapies currently approved by the U.S. Food and Drug Administration to treat fibrosis are for idiopathic pulmonary fibrosis (pirfenidone and nintedanib) [9,10]. Another antifibrotic therapy running phase three clinical trial is also focused on targeting pulmonary fibrosis [11]. Newer inhibitors of TGF-β receptor signaling, including ALK5 inhibitors, are still under investigation as potential antifibrotic agents [12,13,14,15]. However, most drugs designed to treat cardiac inflammation have either failed or have a higher risk of adverse events [16,17,18,19].

We previously identified the cardioprotective effects of a small endogenous tetrapeptide (*N*-acetyl-ser-asp-lys-pro [Ac-SDKP]) that inhibits both macrophage accumulation and collagen formation in the heart in various models of cardiac injury [20,21,22]. Ac-SDKP is a naturally occurring tetrapeptide derived from the enzymatic cleavage of the N-terminal end of thymosin β4 [23]. Despite its robust anti-inflammatory and antifibrotic effects, it was initially unclear how Ac-SDKP inhibited cardiac inflammation fibrosis. In 2006, a seminal publication by Peng et al. [24] demonstrated that the beneficial effects of angiotensin-converting enzyme inhibitors are, at least partly, mediated by Ac-SDKP. While lowering blood pressure is one of the mechanisms through which ACE inhibitors exert their cardioprotective effects, it is not the only factor responsible for their benefits. In our previous study, ACE-transgenic mice with cardiac-specific over expression of ACE showed increased cardiac fibrosis, with normal blood pressure and myocardial Ang II [21]. These animals, however, had significantly decreased Ac-SDKP levels, replacement of which led to the remission of fibrosis, highlighting the cardioprotective effects of Ac-SDKP independent of blood pressure. 

Because Ac-SDKP is a nontoxic endogenous peptide, the discovery of its cardioprotective effects suggested its therapeutic utility for myocardial fibroinflammatory conditions. However, the therapeutic use of Ac-SDKP remained challenging because of its rapid breakdown by angiotensin-converting enzymes, resulting in a short plasma half-life of only 4.5 min [25]. Two research groups independently attempted to stabilize this peptide by synthesizing its analogues, but their biological implications were limited [26,27]. Wang et al. [26] developed a biotinylated Ac-SDKP and tested its antifibrotic effects in a murine model of pulmonary fibrosis. However, its plasma stability and tissue availability were not investigated. Ma et al. [27]. synthesized an Ac-SDKP analogue (Ac-SD_D_K_D_P) that had a serum half-life of 2.5 h, which is of suboptimal value for clinical translation. 

In this study, we report a novel approach to prolong the bioavailability of Ac-SDKP by encapsulating the compound within a stable liposome, resulting in robust cellular uptake and high cardiac biodistribution. This novel formulation was validated and found to be biologically effective in a murine model of acute myocardial infarction (MI).

## 2. Methods

### 2.1. Ac-SDKP Liposome Preparation (L-Ac-SDKP), Purification and Characterization

#### Liposome Composition

Different formulations of liposomes were developed to optimize peptide encapsulation efficiency and cellular uptake. Formulation 1 contained cholesterol (Sigma-Aldrich, St. Louis, MO, USA #C3045) and 1,2-distearoyl-*sn*-glycero-3-phosphocholine (DSPC; Avanti Polar Lipids, Birmingham, AL, USA #850365C) at 30:70. Formulation 2 was composed of cholesterol, DSPC and 1,2-dimyristoyl-*sn*-glycero-3-phospho-*rac*-(1-glycerol) sodium salt (DMPG; Avanti Polar Lipids, Birmingham, AL, USA #840445P) at 30:50:20. Formulation 3 was synthesized using cholesterol (CordenPharma, LIestal, Switzerland #CH-0355), DSPC (CordenPharma, LIestal, Switzerland #LP-R4-076), 1,2-dioleoyl-*sn*-glycero-3-phosphocholine (DOPC; CordenPharma, LIestal, Switzerland #LP-R4-070), and methoxy-poly(ethylene glycol)-1,2-distearoyl-*sn*-glycero-3-phosphoethanolamine-N (DSPE-MPEG; average MW, 2000; CordenPharma, LIestal, Switzerland #LP-R4-039) at 50:33.75:11.25:5 [28,29]. These liposomes were labeled with 0.05% 18:1 Liss Rhod PE (Rh-PE) (1,2-dioleoyl-*sn*-glycero-3-phosphoethanolamine-N-(lissamine rhodamine B sulfonyl) (ammonium salt); Avanti Polar Lipids, Birmingham, AL, USA #810150). Ac-SDKP was synthesized using 9-fluorenylmethoxy carbonyl solid-phase small peptide synthesis (GenScript, Piscataway, NJ, USA) and labeled with fluorescein isothiocyanate (FITC). The purity of Ac-SDKP was >98%. For experiments assessing biological efficacy, FITC and Rh-PE tracers were omitted from the preparation. All other materials were obtained from Sigma-Aldrich, St. Louis, MO, USA.

### 2.2. Liposome Preparation

To generate L-Ac-SDKP, 50 mg of lipids was dissolved in 1 mL of chloroform, and organic solvents were evaporated under gentle nitrogen flow for 2 h. The resulting lipid film was dissolved in 200 µL of ethanol and hydrated using 2 mg/mL Ac-SDKP in phosphate-buffered saline (PBS; pH 7.4) at 60 °C for 10 min. The liposome-peptide solution was sized through a 0.2 μm filter (15 times) using a syringe extrusion method at 60 °C. The resulting mixture was refrigerated for 1 h, facilitating the formation of the liposomes.

### 2.3. Liposome Purification

For purification, a two-step process was employed. First, dialysis in ice-cold PBS at 4 °C with two buffer changes effectively removed the small molecules (e.g., free peptide, ethanol, traces of chloroform) and non-encapsulated components. Following dialysis, size-exclusion chromatography using Sephadex G-25 columns was employed for further purification. The columns were loaded with 1 mL of liposome samples, and liposome-containing fractions were collected. Size-exclusion chromatography isolated liposomes based on size and molecular weight, effectively separating them from contaminants or undesired substances. This process further purified the liposome samples, ensuring the removal of non-encapsulated components. The combined use of dialysis and size-exclusion chromatography resulted in a purified liposome formulation, providing a suitable platform for subsequent experiments and analysis.

### 2.4. Liposome Characterization

Liposome size and zeta potential were measured by dynamic light scattering in a NanoBrook 90Plus phase analysis light scattering instrument as described before [30]. Briefly, sizes and dispersity were measured in PBS, and zeta potential was measured in filtered deionized water. Peptide loading efficiency was characterized by running the samples through a Sephadex G-25 column. The loading efficiency was determined by the amount of drug fluorescence in the liposome-containing fractions [30]. Additionally, the Ac-SDKP concentration was calculated using a commercially available enzyme immunoassay (EIA) kit (see Supplementary Materials).

### 2.5. Cryo-Transmission Electron Microscopy (Cryo-TEM)

Cryo-TEM was performed to examine the ultrastructure of the liposomes with and without loaded Ac-SDKP according to the protocol used by Carter et al. [30] Briefly, lacey carbon grids (Ted Pella, Redding, CA, USA #01890) were treated with chloroform for ~10 s, washed with acetone for 10 s, then isopropyl alcohol for 10 s, and glow discharged at 25 mA for 15 s before the sample was applied. Ac-SDKP-containing or empty control liposomes at a concentration of ~0.2 µg/µL (lipid) were diluted 10× with water. Approximately 3 μL of the diluted preparation was deposited on the electron microscopy grid. Vitrification of the samples was performed in a Vitrobot (FEI) by blotting the grids once for 7 s and with an offset of 10 before they were plunged into liquid ethane. The temperature and relative humidity during the vitrification process were maintained at 25 °C and 100%, respectively. The grid was loaded into the FEI Tecnai F20 electron microscope that operated at 200 kV using a Gatan 626 single-tilt cryo-holder. Images were collected with a TVIPS F416 camera at a nominal magnification of ×25,000, which produced images with a calibrated pixel size of 1.45 Å. 

### 2.6. Fluorescence Confocal Microscopy and Flow Cytometry for Liposome Uptake In Vitro

Cellular uptake of L-Ac-SDKP was assessed in mouse cardiac fibroblasts and macrophages. Briefly, cultured macrophages (RAW 264.7 cells, ATCC) and primary mouse cardiac fibroblasts [20] were incubated with L-Ac-SDKP or free peptides (Ac-SDKP) at a concentration of 0.24 µg/mL. For the intracellular distribution assay, cells were cultured on sterile coverslips, incubated with Ac-SDKP or L-Ac-SDKP for 4 h, fixed with 4% paraformaldehyde, mounted, and imaged using a fluorescence microscope (Zeiss LSM 800 laser scanning microscope) at ×400 magnification. For quantification of the peptide uptake, cells were detached and examined with a BD Accuri C6-Plus flow cytometer and further analyzed by FCS Express 6-De Novo software, version 5.

### 2.7. Cell Proliferation and Toxicity Assay

To access cell viability and proliferation in response to the liposome-peptide, Thiazolyl Blue Tetrazolium Bromide (MTT; Sigma-Aldrich, M2128) assay was performed. Briefly, human cardiac fibroblasts (Lifeline Cell Technology, San Diego, CA, USA #FC-0060) and RAW macrophages were plated into 96-well plates at a density of 8000–10,000 cells/well. After 24 h, cells were serum-starved overnight, followed by incubation in empty liposomes and PBS (L-PBS), L-Ac-SDKP (0.24 µg/mL), or Ac-SDKP (0.24 µg/mL) for an additional 24 h. After this, the media were replaced, and the cells were incubated in the MTT substrate for 3.5 h at 37 °C. The yellow-orange colored product, a stable soluble formazan, was quantified at 450 nm using a microplate reader. Relative viability was calculated in relation to the cells treated with culture media alone as a control.

### 2.8. L-Ac-SDKP in Lipopolysaccharide (LPS)-Induced Macrophage Activation

To investigate the effects of L-Ac-SDKP on macrophage function, we stimulated RAW 264.7 macrophages with *LPS*. Approximately 0.2 × 10^6^ cells were seeded in a 12-well plate and grown for 24 h. The cells were serum-deprived overnight, followed by incubation with 50 ng/mL LPS with and without L-Ac-SDKP (0.24 µg/mL) or Ac-SDKP (0.24 µg/mL) at 37 °C. After 24 h, cells were detached and lysed in TRIzol reagent (Thermofisher Scientific, Grand Island, NY, USA #15596018) for the mRNA isolation. Different inflammatory gene (*Arginase 1*, *Ccl2*, *Lgals3*, *Il1b*, *Il6*, *Nos2*, *Tnfa*, and *18S*) expression levels were determined with quantitative real-time PCR.

### 2.9. L-Ac-SDKP in TGFβ1-Induced Cardiac Fibroblast Activation

Human cardiac fibroblasts were seeded at a density of 0.2 × 10^6^ in a 12-well plate and cultured in human cardiac fibroblast medium (Cell Application, San Diego, CA, USA #316-500). After 24 h, cells were rinsed and cultured in serum-free medium (Lifeline Cell Technology, San Diego, CA, USA #LL0001) for 16 h. Then, the cells were incubated with fresh human cardiac fibroblast medium containing 2.5% FBS with and without TGFβ1 (50 ng/mL) and co-treated with L-Ac-SDKP (0.24 µg/mL) simultaneously. After 72 h, cells were lysed in TRIzol reagent for the mRNA isolation. The gene expression of Acta2, Col1a1, Col3a1, Lgals3, Il4, Mmp9 and 18S was determined with quantitative real-time PCR.

### 2.10. Animal Studies

Twelve- to fifteen-week-old male and female wild-type C57BL/6J mice (The Jackson Laboratory, Bar Harbor, ME, USA #0664) were used for in vivo studies. The animal care and experimental protocols were carried out in accordance with the US National Institutes of Health guidelines and were approved by the Institutional Animal Care and Use Committees of the Roswell Park Comprehensive Cancer Center and University at Buffalo. A total of 22 male and female mice were used for in vivo biodistribution, and 48 mice were used for evaluating the biological efficacy of L-Ac-SDKP. Mice were anesthetized with 2–3% isoflurane inhalation during chest radiation and induction of MI. Additional anesthetics, including ketamine (1 mg/kg intramuscular) and xylazine (5 mg/kg subcutaneous), were given during the MI procedure. Ethiqa XR (3.25 mg/kg) and 0.5 mL of normal saline were injected for post-procedural pain control and rehydration after MI. Mice were euthanized using CO_2_ overexposure and cervical dislocation.

### 2.11. Non-Invasive IVIS Spectrum Optical Imaging and Enzyme Immunoassay for Liposome Biodistribution

For biodistribution and bioavailability studies, a mouse model of cardiac irradiation was used. The left hemithorax regions of animals were exposed to cumulative 45 Gy of radiation (30 Gy and 15 Gy, 7 days apart). Beginning on day 8, animals received either vehicle or L-Ac-SDKP for 3 consecutive days, and a fourth injection was given 4 days later. The animals were euthanized 24 h after the last injection, and organs were harvested for Ac-SDKP measurements as described before [31]. EIA was performed using a commercially available kit (Cayman Chemical, Ann Arbor, MI, USA #589451). 

The biodistribution of the fluorescent compound was analyzed using the IVIS Spectrum in vivo imaging system (PerkinElmer) in a smaller subset of animals exposed to single dose chest radiation as described before [20]. Mice were given four daily doses of the vehicle (PBS), Ac-SDKP (unlabeled) or L-Ac-SDKP (Rh-PE labelled) intraperitoneally (i.p.) beginning 24 h after thoracic irradiation. Mice without chest radiation exposure were used as controls. After completion of the study, the mice were euthanized, and their organs were harvested for additional ex vivo imaging. 

### 2.12. L-Ac-SDKP Biological Efficacy

A group of mice received i.p. injections of L-Ac-SDKP (2.4 mg/kg of body weight every 24 h for 3 days before MI surgeries, immediately after the surgery, and every fourth day subsequently for a total of 5 weeks). A separate subset of mice was treated with continuous infusion of Ac-SDKP (3.6 mg/kg body weight/day) administered via an ALZET osmotic minipump (Durect) as described before [32]. Treatment was started 1 week before MI surgery and continued for 5 weeks post-surgery, similarly to that described in the protocol used before [33]. At the end of the experiment, the survival data were recorded, cardiac function was evaluated, and heart tissues were collected for histological and molecular analyses. 

A transthoracic echocardiogram was used to evaluate cardiac structure and function in lightly sedated (1–2% isoflurane inhalation) mice as described before [34]. Echocardiographic images were acquired using the Vevo 2100 high-frequency small-animal ultrasound system (FUIJIFILM VisualSonics, Inc., Toronto, ON, Canada) with a 55-MHz linear array transducer. The left ventricle (LV) was imaged in anterior short-axis views in the two-dimensional B-mode to determine the dimensions. Heart rates were maintained at ~400–500 bpm. All images were acquired using the highest possible frame rate (233–401 frames/s) depending on the imaging axis to achieve the best possible image resolution. LV dimensions and wall thicknesses were determined from M-mode images at the mid-papillary muscle level, as described previously [35,36]. The basic measurements were obtained using Vevo Lab software 5.7.1, FUJIFILM VisualSonics, Inc. Toronto, ON, Canada. 

Macrophage infiltration was evaluated with immunohistochemical analyses of sections from formalin-fixed paraffin-embedded tissues; collagen deposition was also assessed with Masson’s trichrome staining (Supplementary Materials). 

### 2.13. Quantitative Real-Time PCR

Quantitative real-time PCR was performed to examine the fibroinflammatory gene profile in cultured macrophages and cardiac fibroblasts treated with L-Ac-SDKP and in the heart after L-Ac-SDKP injections in mice with MI. mRNA was extracted using an E.Z.N.A. Total RNA Kit I (Omega Bio-tek, Norcross, GA, USA #R6934-01). cDNA was synthesized using SuperScript III reverse transcriptase (Thermo Fisher, Grand Island, NY, USA #18080093). Quantitative real-time PCR was performed using SsoFast EvaGreen Supermix (Bio-Rad, Hercules, CA, USA #1725201) for genes associated with fibroinflammatory pathways (including those encoding *Acta2*, *Arginase 1*, *Ccl2*, *Cd38*, *Col1a1*, *Col3a1*, *Egr2*, *Lgals3*, *Il1b*, *Il4*, *Il6*, *Il10*, *Mmp9*, *Nos2*, *Pparg*, *Stat6*, *Tgfb1*, *Tnfα*, and *18S*). Primer sequences used for SYBR green-based RT-PCR are provided in Supplementary Table S2. Samples were run in triplicates, and gene expression was calculated by threshold cycle analysis using 18S as a reference. 

### 2.14. Statistical Analyses

Quantitative endpoints were summarized by group using the means and standard deviations. Two-group comparisons were made using unpaired *t* tests for equal variance. Comparisons among more than two groups were performed via ANOVAs using the log-transformed data, with Tukey adjusted post hoc comparisons between individual treatment groups. All statistics were performed using SAS version 9.4. *p* values of <0.05 were considered significant.

## 3. Results 

### 3.1. Optimization and Characterization of Liposomes

To obtain liposomes with both higher cellular uptake and increased loading efficiency, saturated and unsaturated lipids were blended at different molar ratios. Formulation 1 containing a lipid mixture of cholesterol and DSPC had a very low peptide loading efficiency (2.5% ± 0.5%). Formulation 2 contained anionic lipid DMPG in addition to cholesterol and DSPC and had a peptide loading efficiency of 1.5% ± 0.5% (see Figure S1). Although the second formulation showed higher cellular uptake in the macrophages, the encapsulation efficiency remained very low. In formulation 3, the liposome composition was modified to include cholesterol, DSPC, DOPC, and DSPE-MPEG. This resulted in a higher peptide uptake efficiency (up to 7 times higher than for the first two formulations, Figure S1). The steric modification with the addition of MPEG is known to prevent rapid plasma clearance by reticuloendothelial cells and facilitate the in vivo retention of liposomes [37,38]. Formulation 3 was, therefore, used in all subsequent analyses. 

### 3.2. Structural Homogeneity of Ac-SDKP-Containing Liposomes

**Figure 1:**
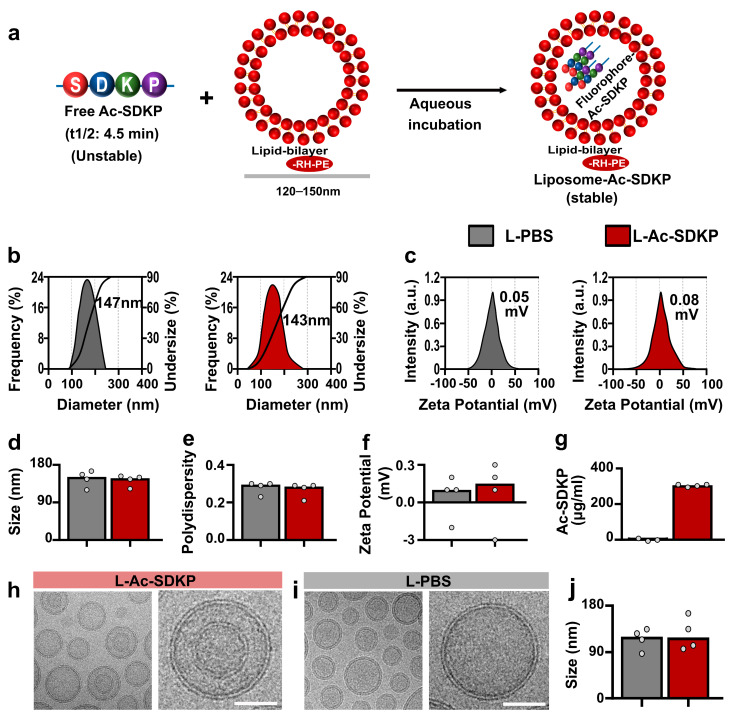
**Liposome characterization**. (**a**) Schematic representation of free Ac-SDKP, a liposome (a lipid bilayer nanoparticle), and L-Ac-SDKP (Ac-SDKP inside the liposome). (**b**,**c**) Representative graphs of liposome size distribution and zeta potential, respectively, by nanoparticle tracking analysis. Comparative data for empty liposome (L-PBS) and L-Ac-SDKP for (**d**) size, (**e**) polydispersity, and (**f**) zeta potential. (**g**), Ac-SDKP concentration in the liposomal formulations (L-PBS vs. L-Ac-SDKP). Cryo-TEM images of L-Ac-SDKP (**h**) and empty liposome (L-PBS) (**i**) and a comparative data for size (**j**). Scale bars, 60 nm.

The hydrodynamic diameters of liposomes loaded with Ac-SDKP (schematic view Figure 1a) were morphologically homogeneous, with sizes ranging from 95 to 250 nm and peaking at 140 to 160 nm (Figure 1b). The zeta potential of loaded liposomes was near neutral (0.075 ± 0.26 mV, *n* = 4) (Figure 1c). There were no differences in the zeta potential, size, or polydispersity of the liposomes with and without Ac-SDKP loading (Figure 1d–f). These parameters are important for particle stability and influence the in vivo fate of the liposomes. The Ac-SDKP concentration in the liposome was 303.67 ± 6.27 µg/mL as measured by EIA (Figure 1g). Cryo-TEM revealed no differences in the shapes of free liposomes and those loaded with Ac-SDKP, but Ac-SDKP-loaded liposomes showed aggregates in the core. The mean vesicle diameter of L-Ac-SDKP measured on cryo-TEM was 125 ± 27.40 nm (Figure 1h–j). 

### 3.3. Enhanced Uptake of L-Ac-SDKP by Cultured Cells

**Figure 2:**
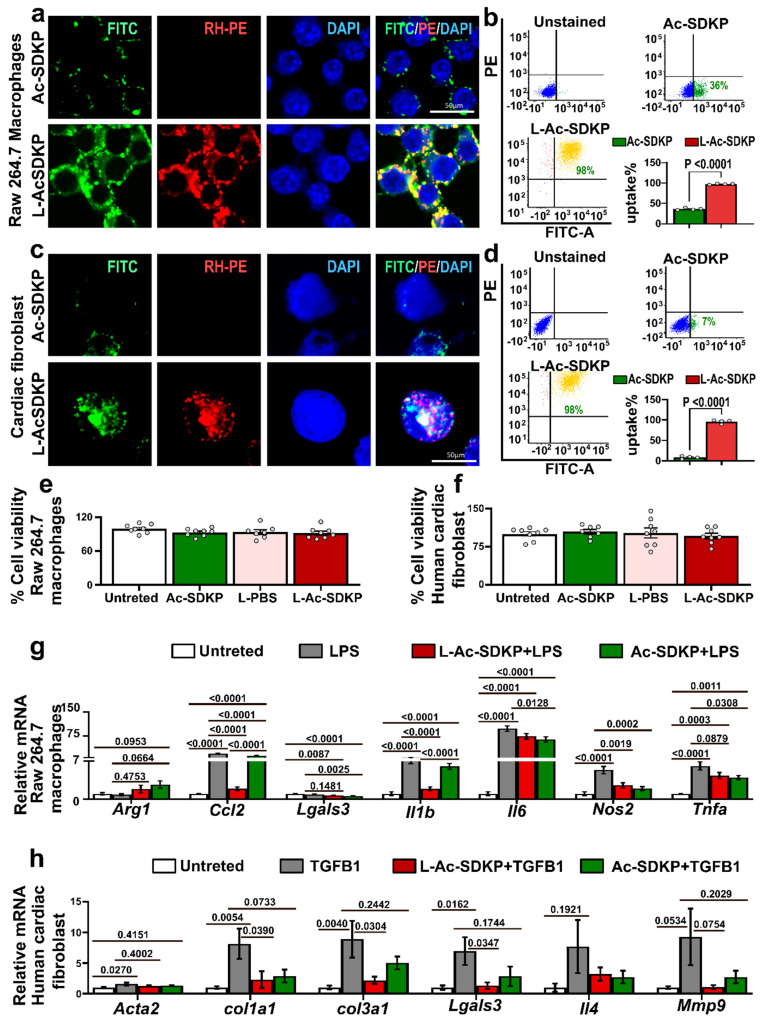
**In vitro uptake of free Ac-SDKP and L-Ac-SDKP**. RAW 263.2 macrophages (**a**,**b**) and primary mouse cardiac fibroblasts (**c**,**d**) were incubated with FITC-labeled Ac-SDKP (0.24 µg/mL) with or without liposomes (labeled with Rh-PE) for 4 h. Colocalization of the Ac-SDKP and liposomes was determined by the presence of FITC and Rh-PE positivity. Representative fluorescence images of FITC (green), Rh-PE (red), and DAPI (blue) and the composite demonstrating colocalization of the Ac-SDKP and liposomes within the cells with perinuclear accentuation within the macrophages (**a**) and nuclear localization within the fibroblasts (**c**). Scale bar, 50 μm. Representative flow cytometry results corresponding to Ac-SDKP (FITC, green) and L-Ac-SDKP (FITC + Rh-PE, yellow) uptake by macrophages (**b**) and fibroblasts (**d**). The gating was set to show the FITC^+^ Rh-PE^−^ cell population in green, FITC^−^ Rh-PE^+^ population in red, and FITC^+^ Rh-PE^+^ population in yellow; FITC^−^ Rh-PE^−^ (viable cell nuclei) cells are in blue. (**e**,**f**) show percent cell viability in raw macrophages and human cardiac fibroblasts with empty liposome (L-PBS), Ac-SDKP (0.24 ug/mL), and L-Ac-SDKP treatment (*n* = 7–8 in each treatment). (**g**) represents relative expression levels of genes mostly involved in inflammatory process in the raw macrophages induced by LPS, with and without Ac-SDKP or L-Ac-SDKP (*n* = 8–9). (**h**) shows relative expression levels of genes predominantly involved in fibrotic processes in cardiac fibroblasts induced by TGFβ1, with and without Ac-SDKP or L-Ac-SDKP (*n* = 8–10).

Fluorescence microscopy demonstrated a robust uptake of L-Ac-SDKP by macrophages and cardiac fibroblasts (Figure 2a,c) after 4 h of treatment. The Rh-PE plus FITC-labeled L-Ac-SDKP localized mainly in the nuclear and perinuclear cytoplasmic spaces. Additional flow cytometry analysis was performed to quantify the percentage of FITC-positive macrophages and fibroblasts, showing significantly higher uptake by L-Ac-SDKP-treated macrophages (Ac-SDKP, 36.5% ± 2.29%; L-Ac-SDKP, 97.5% ± 1.12%; *p* < 0.0001, *n* = 4) and cardiac fibroblasts (Ac-SDKP, 8.5% ± 2.96%; L-Ac-SDKP, 95.75% ± 3.96%; *p* < 0.0001, *n* = 4) within 4 h of treatment (Figure 2b,d). Multiple doses of L-Ac-SDKP were tested, and the highest uptake was detected with the 0.24 µg/mL concentration.

### 3.4. Effects of L-Ac-SDKP on Cell Survival and Gene Expression Profile in Cultured Macrophages and Cardiac Fibroblasts

We examined the effects of L-Ac-SDKP on cell viability by MTT assay. We treated RAW 264.7 macrophages and human cardiac fibroblasts with Ac-SDKP (with and without liposome encapsulations) for 24 h. The treatment of free or Liposomal Ac-SDKP did not affect percent cell viability of both macrophages and cardiac fibroblasts at 0.24 µg/mL of Ac-SDKP concentration (Figure 2e,f). 

To investigate the effects of L-Ac-SDKP on inflammatory genes, macrophages were stimulated with 50 ng/mL LPS, with and without L-Ac-SDKP. LPS treatment induced the gene expression level of *Monocyte Chemoattractant Protein-1* (MCP1/*Ccl2*, *p* < 0.0001 vs. untreated; *n* = 8–9), interleukin (IL)-1β (*Il1b*, *p* < 0.0001 vs. untreated; *n* = 8–9), interleukin (IL)-6 (*Il6*, *p* < 0.0001 vs. untreated; *n* = 8–9), nitric oxide synthase 2 (*Nos2*, *p* < 0.0001 vs. untreated; *n* = 8–9), and tumor necrosis factor alpha (*Tnfa*, *p* < 0.0001 vs. untreated; *n* = 8–9) but did not alter the expression of Arginase 1 (*Arg1*) or Galectin 3 (*Lgals3*). Co-treatment with L-Ac-SDKP significantly decreased the mRNA levels of MCP1/*Ccl2* (*p* < 0.0001 vs. LPS; *n* = 8), *Il1b* (*p* < 0.0001 vs. LPS and *p* < 0.0001 vs. Ac-SDKP + LPS; *n* = 8), and *Nos2* (*p* = 0.0019 vs. LPS; *n* = 8). Additionally, L-Ac-SDKP treatment showed a trend to reduce the mRNA expression of *Il6* (*p* = 0.1094 vs. LPS; *n* = 8), *Lgals* (*p*= 0.1481 vs. LPS; *n* = 8) and *Tnfa* (*p* = 0.0879 vs. LPS; *n* = 8) (Figure 2g).

We examined the effects of L-Ac-SDKP on the mRNA expression profile of cardiac fibroblasts stimulated with TGFβ1. After 72 h, TGFβ1 treatment increased the expression of smooth muscle alpha actin (*Acta2*, *p* = 0.0270 vs. untreated; *n* = 8–10), Collagen 1 (*Col1a1*, *p* = 0.0054 vs. untreated; *n* = 8–10), Collagen 3 (*Col3a1*, *p* = 0.0040 vs. untreated; *n* = 8–10), Galectin 3 (*Lgals3*, *p* = 0.0162 vs. untreated; *n* = 8–10), and matrix metallopeptidase 9 (*Mmp9*, *p* = 0.0534 vs. untreated; *n* = 8–10), while the expression of interleukin (IL)-4 was not changed (*Il4*, *p* =0.1921 vs. untreated; *n* = 8–10). L-Ac-SDKP co-treatment significantly reduced the expression of *Col1a1* (*p* = 0.0390 vs. TGFβ1; *n* = 8), *Col3a1* (*p* = 0.0304 vs. TGFβ1; *n* = 8), *Lgals3* (*p* = 0.0347 vs. TGFβ1; *n* = 8) and showed a tendency to lower the expression of *Mmp9* (*p* = 0.0754 vs. TGFβ1 only; *n* = 8). The levels of *Acta2* (*p* = 0.4002 vs. TGFβ1 only; *n* = 8) and *Il4* (*p* = 0.5659 vs. TGFβ1 only; *n* = 8) genes were not altered with L-Ac-SDKP (Figure 2h).

### 3.5. Biodistribution and Bioavailability of Ac-SDKP after i.p. Administration

**Figure 3:**
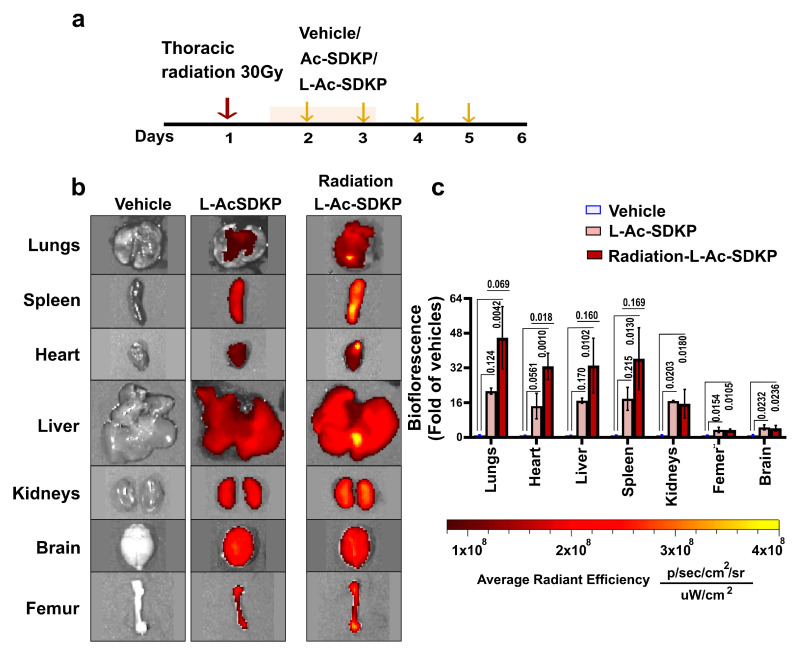
L-Ac-SDKP bioavailability after daily i.p. injection in mice with and without left chest radiation. Biodistribution of Ac-SDKP in the serum and heart was evaluated after four i.p. injections given daily one day after thoracic irradiation. (**a**), representative IVIS spectrum images (**b**), and biofluorescence quantification (**c**) of the tissues demonstrating fluorescence intensity in animals receiving L-Ac-SDKP with and without radiation normalized to that in PBS-treated (vehicle) controls. The coloring corresponds to the spectrum gradient bar for the radiant efficiency unit (p/s/cm^2^/sr). Scale bar, 5 mm.

The biodistribution of L-Ac-SDKP was assessed after repeated i.p. injections in mice with or without irradiation. Two sets of experiments were performed; in the first set of experiments, we sought to examine the cardiac uptake of fluorescence-labelled L-Ac-SDKP with ex vivo imaging in control and irradiated mice after four i.p. injections given 24 h apart (Figure 3a,b). L-Ac-SDKP was started 24 h after thoracic radiation (30 Gy, single fraction). Mice given PBS were used as controls to normalize the biofluorescence in the liposome-treated hearts. Biofluorescence in irradiated mice treated with L-Ac-SDKP was higher than the control mice treated with L-Ac-SDKP (biofluorescence fold change: L-Ac-SDKP, 14.41 ± 4.16 vs. radiation L-Ac-SDKP, 32.69 ± 4.97; *p* = 0.018, *n* = 2–3). In addition to the heart, repeated i.p. injections resulted in the accumulation of L-Ac-SDKP in the lungs, liver, kidneys, femur, and brain.

**Figure 4:**
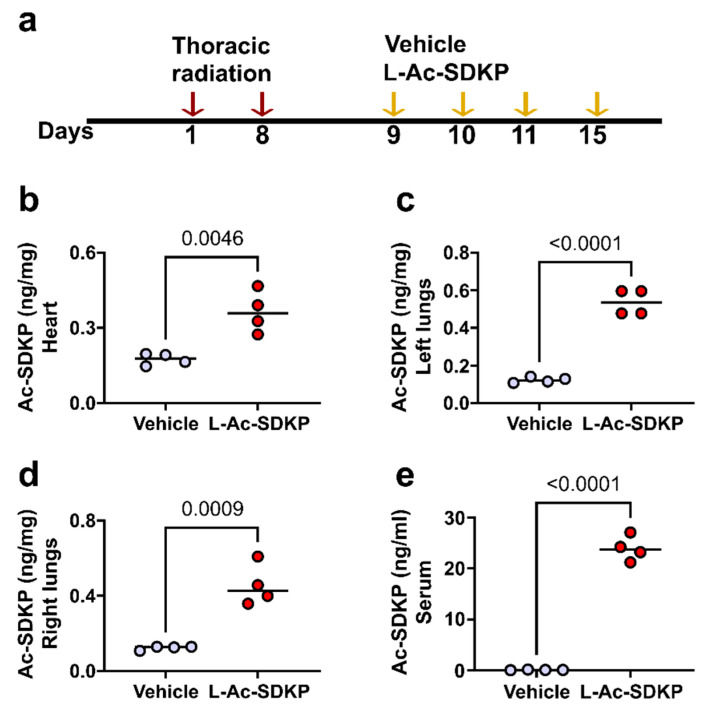
Cardiac tissue and serum bioavailability after L-Ac-SDKP i.p. injection at intermittent dose in mice after 45 Gy chest radiation. Experimental timeline for data shown in panel (**a**). (**b**–**e**) Ac-SDKP levels in tissue homogenates (heart, left lungs, right lungs and serum) from irradiated mice treated with vehicle (PBS) or L-Ac-SDKP (*n* = 4) after three daily injections followed by a fourth dose 4 days later.

The second set of experiments was performed to quantify the serum and tissue levels of Ac-SDKP when administered with and without liposome encapsulation. For these experiments, mice received irradiation to the left hemithorax (two fractions, 30 Gy and 15 Gy, 1 week apart) and three consecutive i.p. injections of L-Ac-SDKP, 24 h apart. The fourth dose was administered 4 days later, and the animals were sacrificed 24 h after the last injection (Figure 4a). PBS was administered to control animals. Repeated i.p. injections of L-Ac-SDKP at this interval of administration increased the levels of Ac-SDKP in various organs, including the heart, and lungs (*p* < 0.05, *n* = 4) (Figure 4b–e).

### 3.6. Effects of L-Ac-SDKP on Cardiac Function after Acute MI

**Figure 5:**
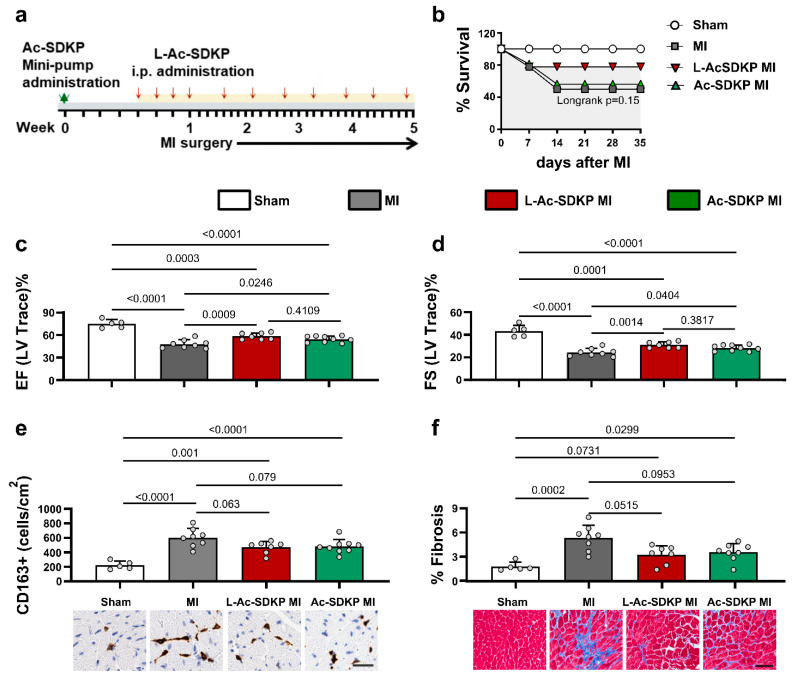
**Effects of L-Ac-SDKP on cardiac function, macrophage infiltration, and fibrosis in mice after acute MI.** C57BL/6J mice received Ac-SDKP or L-Ac-SDKP via i.p. injections. (**a**) Experimental timeline. (**b**) Survival curves of mice without MI, with MI, and with MI treated with L-Ac-SDKP or the free peptide. Echocardiography analyses of ejection fraction (EF) (**c**) and fractional shortening (FS) (**d**) in the L-Ac-SDKP- and Ac-SDKP-treated mice compared to sham and untreated control mice (*n* = 5–9). (**e**) Immunohistochemical analysis of LV sections showing infiltration of CD163^+^ macrophages in experimental mice (*n* = 5–9). Scale bar, 20 µm. (**f**) Interstitial collagen volume fractions and quantitative data of fibrosis in experimental mice (*n* = 5–8). Scale bar, 20 µm.

The biological effects of the newly developed L-Ac-SDKP were evaluated in a mouse model of acute MI. Mice were given either continuous infusion of free unlabeled Ac-SDKP through ALZET osmotic minipumps or intermittent i.p. injections of unlabeled L-Ac-SDKP as described in Figure 5a. All mice included in the study survived for 24 h, but a subset of mice died between day 2 and day 14 after acute MI. The survival rate of mice in the MI controls was 44% (8/18), whereas the survival of L-Ac-SDKP-treated MI animals was 78% (7/9) and that of Ac-SDKP-treated MI mice was 56% (9/16) (Figure 5b).

Echocardiography analysis was performed 5 weeks after MI, immediately prior to the terminal experiment. The induction of acute MI significantly decreased the ejection fraction (EF) compared to that in the sham controls (sham, 74.90 ± 4.98%; MI, 47.98 ± 5.30%; *p* < 0.0001; *n* = 5–8). Treatment with both free and L-Ac-SDKP improved EF in animals with MI (L-Ac-SDKP, 58.47 ± 4.03% (*p* = 0.0009 vs. MI only); Ac-SDKP, 54.54 ± 3.90% (*p* = 0.0246 vs. MI only), *n* = 7–9; Figure 5c). Similarly, fractional shortening (FS) was severely decreased after MI (24.39 ± 3.31%), which was significantly improved by both free Ac-SDKP and L-Ac-SDKP treatments (L-Ac-SDKP, 30.99 ± 2.48% (*p* = 0.0014); Ac-SDKP, 28.31 ± 2.51% (*p* = 0.04); *n* = 7–9; Figure 5d). Other functional parameters assessed by echocardiography, including LV end systolic and end diastolic diameters, volumes, stroke volume, and LV mass, were not altered by free or L-Ac-SDKP treatment (Table S2).

### 3.7. Effects of L-Ac-SDKP on Myocardial Fibrosis and Macrophage Infiltration after Acute MI

Immunohistochemical analysis of the LV sections showed markedly increased infiltration of CD163^+^ macrophages in the myocardium of mice with MI. Macrophages were present in clusters in the border zone, but countable dispersed individual cells were observed in the remote zone (sham, 222.80 ± 49.89 CD163^+^ cells/cm^2^; MI, 602.13 ± 118.24 CD163^+^ cells/cm^2^; *p* < 0.0001; *n* = 5–8). The hearts of mice treated with L-Ac-SDKP tended to have fewer CD163^+^ cells than those from vehicle-treated MI mice, but the difference was not statistically significant (L-Ac-SDKP, 468.57 ± 77.90 CD163^+^ cells/cm^2^; *p* = 0.063 vs. MI only; *n* = 7–8). Similar results were obtained when comparing Ac-SDKP- and vehicle-treated mice with MI (*p* = 0.079; *n* = 8–9; Figure 5e). 

To examine the effects of L-Ac-SDKP treatment on cardiac fibrosis, the collagen volume fraction was calculated in the remote zone of the myocardial sections after Masson’s trichrome staining. Compared to the amount of interstitial fibrosis in vehicle-treated mice with MI (5.33 ± 1.46%), fibrosis was decreased in L-Ac-SDKP-treated animals with MI (L-Ac-SDKP, 3.23 ± 1.06% (*p* = 0.0515; *n* = 7–8); Figure 5f). 

### 3.8. Effects of L-Ac-SDKP on Fibroinflammatory Gene Expression Profile

**Figure 6:**
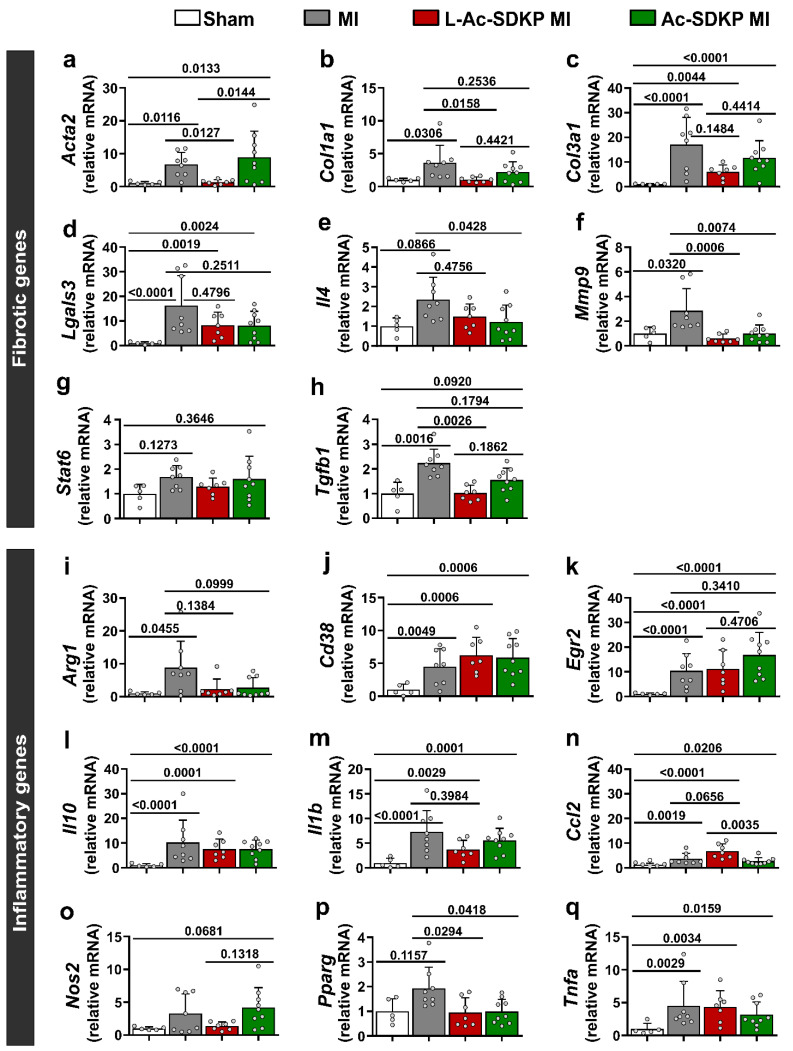
**Effects of L-Ac-SDKP on fibroinflammatory gene changes 5 weeks after MI.** Relative expression levels of genes predominantly involved in fibrotic (**a**–**h**) and inflammatory (**i**–**q**) processes in the myocardial tissues of mice without MI and those with MI untreated or treated with L-Ac-SDKP or Ac-SDKP (*n* = 5–9, L-Ac-SDKP MI vs. Ac-SDKP MI).

The increase in cardiac fibrosis induced by MI was paralleled by significantly increased expression of the profibrotic genes, including those for *Acta2* (6.71 [±3.45]-fold of the control; *p* = 0.0116 vs. sham; *n* = 5–8), *Col1a1*, 3.62 [±2.47]-fold of the control; *p* = 0.0306 vs. sham; *n* = 5–8, *Col3a1*, 17.08 [±10.30]-fold of the control; *p* < 0.0001 vs. sham; *n* = 5–8, *Lgals3*, 16.29 [±11.31]-fold of the control; *p* < 0.0001 vs. sham; *n* = 5–8, *Mmp9*, 2.85 [±1.68]-fold of the control; *p* = 0.0320 vs. sham; *n* = 5–8, and *Tgfb1*, 2.24 [±0.53]-fold of the control; *p* = 0.0016 vs. sham; *n* = 5–8, as shown in Figure 6a–d,f,h, respectively. Treatment with L-Ac-SDKP significantly reduced the expression of *Acta2* (1.34 [±0.70]-fold of the control; *p* = 0.0127 vs MI; *n* = 7–8), *Col1a1* (1.05 [±0.38]-fold of the control; *p* = 0.0158 vs. MI; *n* = 7–8), *Mmp9* (0.61 [±0.32]-fold of the control; *p* = 0.0006 vs. MI; *n* = 7–8), and *Tgfb1* (1.03 [±0.29]-fold of the control; *p* = 0.0026 vs. MI; *n* = 7–8). The expression of *Acta2* was lower in L-Ac-SDKP-treated mice than in free Ac-SDKP-treated mice (*p* = 0.0144).

Additional gene analysis was performed to understand the expression profile of genes primarily implicated in the inflammatory process. Induction of MI increased the expression of arginase 1 (*Arg1*, 8.86 [±7.48]-fold of the control; *p* = 0.0455 vs. sham; *n* = 5–8), *Cd38* (4.51 [±2.55]-fold of the control; *p* = 0.0049 vs. sham; *n* = 5–8), *Egr2* (10.37 [±6.48]-fold of the control; *p* < 0.0001 vs. sham; *n* = 5–8), interleukin (IL)-10 (*Il10*, 10.21 [±8.46]-fold of the control; *p* < 0.0001 vs. sham; *n* = 5–8), IL-1β (*Il1b*, 7.29 [±4.05]-fold of the control; *p* < 0.0001 vs. sham; *n* = 5–8), MCP1 (*Ccl2*, 3.45 [±2.07]-fold of control; *p* = 0.0019 vs. sham; *n* = 5–8), and tumor necrosis factor alpha (*Tnfa*, 4.53 [±3.48]-fold of the control; *p* = 0.0029 vs. sham; *n* = 5–8) compared to that in the sham controls (Figure 6i–n,p,q). L-Ac-SDKP administration significantly reduced the expression of peroxisome proliferator-activated receptor gamma (*Pparg*, 0.95 [±0.55]-fold of the control; *p* = 0.0294 vs. MI) and slightly reduced the expression (though not significantly) of *Arg1* (*p* = 0.1384 vs. MI) compared to that in vehicle-treated mice with MI (Figure 6i,p). The levels of *Cd38*, *Egr2*, *Il1b*, *Il10*, *Nos2* (encoding nitric oxide synthase 2), and *Tnfa* were not altered (Figure 6j–m,q).

## 4. Discussion

Previous studies from our group and other investigators have reported the cardioprotective effects of Ac-SDKP in various models of cardiovascular injury. However, the therapeutic translation of Ac-SDKP remained a challenge because of its short half-life in plasma. We report a novel, stable lipid nanoparticle-bound Ac-SDKP that has robust cellular and tissue uptake and superior biological efficacy over that of free Ac-SDKP. The stable L-Ac-SDKP compound was readily taken up both by cardiac fibroblasts and macrophages. Importantly, the modified liposomal compound showed higher plasma and tissue bioavailability than the free micropeptide after parenteral (intravenous or intraperitoneal) administration and was taken up more robustly by injured myocardium after cardiac radiation. Our newly developed L-Ac-SDKP reduced the myocardial fibroinflammatory response, preserved myocardial function, and improved survival in mice after acute MI.

### 4.1. Liposomes as Vehicles for Stable Ac-SDKP Delivery and Cellular Uptake

Liposomes can be used to stabilize therapeutic compounds, thereby overcoming obstacles to cellular and tissue uptake and improving the biodistribution of compounds in vivo. This enables effective delivery of encapsulated compounds to target sites while minimizing off-target toxicity [39]. Indeed, the use of liposomal nanoparticles as a vehicle to deliver Ac-SDKP protected it from rapid proteolytic degradation, thereby improving its uptake by cells. 

Conventional liposomal formulations are prone to non-specific uptake by tissue-fixed macrophages and other cells belonging to the reticuloendothelial system. This nonspecific immune uptake can sometimes lead to the rapid elimination of Ac-SDKP from the bloodstream, thereby limiting its therapeutic efficacy [40,41,42]. Different techniques have been utilized to stabilize liposomes with superior function, including a novel technique using lipid-polyphosphocholine conjugates [43] and polyethylene glycol chains (PEGylation). We used a polyethylene glycol (PEG) base to sterically stabilize the liposomes. The establishment of a steric barrier improves the efficacy of encapsulated agents by reducing in vivo opsonization with serum components and the rapid recognition and uptake by the reticuloendothelial system [37,38,44]. The protein encapsulation depends on electrostatic interactions between the protein/peptide peripheral surface and the polar head group of phospholipids [45]. Formulation 1 containing a lipid mixture of cholesterol and DSPC had a very low peptide loading efficiency. Formulation 2 contained anionic lipid DMPG in addition to cholesterol and DSPC, which showed higher uptake by macrophages, but the peptide encapsulation efficiency remained very low. We noted that the formulation 3 containing DOPC, DSPC, DSPE-MPEG, and cholesterol had a higher Ac-SDKP loading efficiency than the one containing DMPG, DSPE, and cholesterol. With this formulation, serum levels of L-Ac-SDKP remained steady for up to 24 h after i.p. administration. A similar formulation based on these lipids was previously developed by our group for passive encapsulation of small molecule cargo [28] and was also applicable to protein cargoes [29]. 

The size of a nanoparticle is key for efficient cellular uptake [46] and to reduce potential toxicity to the cells [47]. Moreover, the size also determines the pathway engaged for cellular uptake. Nanoparticles between 120 and 150 nm (but up to 200 nm) are internalized via clathrin- or caveolin-mediated endocytosis [48,49]. The L-Ac-SDKP developed in our lab was ~150 nm in size, and thus expected to be taken up via these endocytosis pathways. The shape of the nanoparticle also contributes to its uptake and intracellular trafficking. Chithrani et al. [50] reported that 5-fold more gold nanoparticles were taken up by HeLa cells when the particles were spherical than when they were rod shaped. Xu and coworkers [51] similarly studied the cellular uptake of FITC-tagged, layered double hydroxide nanoparticles shaped as spheres and rods. Both shapes were taken up via clathrin-mediated endocytosis; however, the nanospheres were retained in the cytoplasm, whereas the nanorods were trafficked toward the nucleus along microtubules. Our L-Ac-SDKP particles are spherical and were observed (via cryo-TEM) in the cytoplasm, consistent with the results reported by Xu and coworkers [51]. 

The fate of nanocarriers is often followed by tracing one or more fluorescent dyes associated with carriers [52]. We applied confocal microscopy and flow cytometry to examine the cellular uptake of the Rh-PE-labeled liposomes and FITC-conjugated Ac-SDKP in vitro. IVIS Spectrum optical imaging, a sensitive tool for detecting fluorescent signals in tissues, was performed to detect and quantify the L-Ac-SDKP in different organs ex vivo. Because the labeling of nanoparticles may alter their physicochemical properties [52,53], we tested unlabeled versions to assess the therapeutic efficacy of L-Ac-SDKP in the MI model.

### 4.2. Model of Radiation-Induced Cardiac Injury for L-Ac-SDKP Biodistribution

We chose the model of radiation-induced cardiac injury instead of the MI model to examine the biodistribution/bioavailability of L-Ac-SDKP. Radiation-induced myocardial injury is a minimally invasive method to induce injury that does not compromise the regional vascular supply. Therefore, we were able to track and quantify the distribution of L-Ac-SDKP in injured myocardium. Notably, myocardial concentrations of L-Ac-SDKP were higher in irradiated mice, demonstrating the preferential uptake of peptide by the injured tissue. Furthermore, the tissue levels of Ac-SDKP were higher in the animals exposed to 45 Gy (two fractions) than in those exposed to 30 Gy (single fraction) radiation, indicating a direct relationship between the degree of tissue injury and L-Ac-SDKP uptake. 

### 4.3. Biological Efficacy of L-Ac-SDKP in an Acute MI Model

The antifibrotic and anti-inflammatory properties of Ac-SDKP have been established in various models of myocardial injury, including hypertensive cardiomyopathy, radiation-induced cardiomyopathy and ischemic cardiomyopathy. We utilized the model of ischemic cardiac injury to examine the biological efficacy of the newly developed liposome-encapsulated Ac-SDKP. This allowed us to test the proof of concept that L-Ac-SDKP, when administered at an intermittent dose, offers at least similar biological effects as the free Ac-SDKP, delivered constantly at a higher dose regimen, in preventing MI-induced myocardial remodeling. The cardioprotective effects of Ac-SDKP in rodent models of cardiac injury have been partly attributed to the inhibition of cardiac fibroblasts and macrophage activity [20,21,22].

Macrophages accumulate in the injured myocardium within 72 h of ischemic cardiac injury or MI and persist for several weeks [54,55]. We previously showed that Ac-SDKP inhibits macrophage differentiation, mobilization, and TNF-α release in vitro [56]. In the present study, we show that L-Ac-SDKP reduces the numbers of macrophages in the myocardium after MI in vivo.

Macrophages display biphasic activation after MI; proinflammatory M1 macrophages peak at 3 days after MI, whereas profibrotic/reparative M2 macrophages peak at 7 days after M [57]. Macrophages activated by the classical pathway (M1 macrophages) secrete proinflammatory and proteolytic cytokines and enzymes such as TNFα, MMPs, IL-1β, and IL-6. By contrast, macrophages following the alternative pathway (M2) secrete extracellular matrix components such as fibronectin and arginase 1 [27] and are important for tissue repair after cardiac ischemic injury [58]. An Ac-SDKP analog, Ac-S_D_DK_D_P, was shown to improve cardiac function while attenuating M2 macrophages after MI [27,59]. In both of our studies, Ac-SDKP did not alter the M1 macrophage profile. In line with these findings, we noted that Ac-SDKP predominantly attenuated the expression profile of fibrosis-related genes such as *Tgfb1*, *Acta2*, and *Mmp9* and inflammation-associated genes *Pparg* and *Arg1* that are largely expressed by M2 macrophages. The effect on gene profiles was similar for the free Ac-SDKP and L-Ac-SDKP; however, the changes were more robust with L-Ac-SDKP. We attribute the stronger effect of L-Ac-SDKP to the higher cellular uptake and the enhanced biological efficiency of the liposomal compound.

In addition to macrophages, fibroblasts and myofibroblasts play active role in chronic myocardial remodeling after MI. Fibrosis composition is tightly regulated by inflammatory and other cell types, paracrine mechanisms (including transforming growth factors), and collagen-degrading enzymes such as matrix metalloproteinases (MMPs). Cytokine stimulation may play a key role in fibroblast activation during the inflammatory phase of cardiac repair. In vitro, IL-1β, and TNF-α promote an inflammatory phenotype in cardiac fibroblasts, inducing cytokine and chemokine synthesis [60,61]. Cytokines also regulate the synthesis of extracellular matrix proteins and modulate matrix metabolism by inducing the expression of matrix-degrading proteases [62,63]. While L-Ac-SDKP show similar patterns of effects on the expression levels of several fibroinflammatory genes, including IL-10, collagen I and collagen III, in vivo and in vitro, a few other genes, such as galectin-3 and MMP-9, showed slightly different results. This could be due to the fact that these genes, including galectin-3, MMP-9 and interleukins, are expressed by both inflammatory cells and fibroblasts and they are regulated by a multitude of cytokines in vivo. The net effects of the peptide on these genes would depend on these factors and the timing of the assay after ischemic injury. Collectively, our in vitro and in vivo data suggest that the cardioprotective effects of Ac-SKDP are primarily related to the modulation of the tissue repair activity of myofibroblasts and macrophages rather than the inhibition of solely macrophage-related inflammatory responses.

The effects on post-MI cardiac fibrosis as well as the expression of several fibroinflammatory gene profile, including those encoding *ACTA2*, *Arginase 1*, *CCL2*, *CD38*, *Col1A1*, *Col3A1*, *EGR2*, *Lgals3*, *IL1B*, *IL4*, *IL6*, *IL10*, *MMP9*, *NOS2*, *PPARγ*, *STAT6*, *TGFβ1* and *TNF-α*, were similar with L-Ac-SDKP compared to free Ac-SDKP both in vitro and in vivo. This showed that liposomal-encapsulated Ac-SDKP offered similar biological efficacy and cardioprotection while remaining stable in the circulation system, allowing intermittent dosing.

## 5. Limitations

Our study proves the concept that liposome-encapsulated Ac-SDKP offers biological efficacy comparable to unencapsulated Ac-SDKP in chronic myocardial remodeling following ischemic cardiac injury. As peptide therapy was initiated before the onset of the MI in this study, further studies will be necessary to mimic therapeutic intervention after acute MI. Similarly, the study period of 5 weeks was designed to examine the effects of L-Ac-SDKP on chronic myocardial remodeling after MI. Examination of the cardiac inflammatory profile at earlier time points would provide a better insight into its effects on macrophage activity. Our goal was to compare the biological efficacy of the newly developed formulation at a comparable tissue level achieved through free peptide administration. Additional study using a similar route of administration for both free and encapsulated peptides could potentially demonstrate the superior efficacy of the novel formulation.

## 6. Conclusions

The cardioprotective effects of Ac-SDKP have been established in various preclinical models of myocardial injury. However, Ac-SDKP is rapidly degraded in the circulatory system by endopeptidase, requiring continuous infusion to maintain a therapeutic level. This has been a major hurdle in the clinical use of this peptide. We developed a stable liposome-encapsulated Ac-SDKP compound that can be delivered via an intermittent parenteral regimen to achieve high myocardial tissue concentrations with robust biological effects. Our data suggest that L-Ac-SDKP offers similar, if not superior, effects as free Ac-SDKP in inhibiting the fibroinflammatory response and improves survival in preclinical models of ischemic cardiac injury. The present research provides a sound basis for further translational studies and future clinical trials to treat subjects with ischemic myocardial injury and fibrotic myocardial remodeling.

## Data Availability

The data presented in this study are available on request from the corresponding author.

## Supplementary Materials

The following supporting information can be downloaded at: https://www.mdpi.com/article/10.3390/cells12121589/s1, Figure S1. Optimization of liposomal formulations. Ac-SDKP encapsulation (loading) efficiency of three different liposomal formulations (#1, #2, and #3). FITC was conjugated to both free and L-Ac-SDKP. The loading efficiency was calculated according to the fluorescence intensity relative to a known concentration of Ac-SDKP. Table S1. Echocardiogram data. Table S2. Primers sequences.
