# Supplementary material for: Phospholipid Encapsulation of an Anti-Fibrotic Endopeptide to Enhance Cellular Uptake and Myocardial Retention
Source: Cells. 2023 Jun 8;12(12):1589. doi: 10.3390/cells12121589 (PMC10296995; doi:10.3390/cells12121589)
Supplement: Supplementary file 1 [file cells-12-01589-s001.zip › cells-2404859-supplementary.pdf]

Supplemental material:

Method:

Ac-SDKP enzyme immunoassay:

Ac-SDKP concentration was measured in prepared liposomes and different mouse tissues including heart, right and left lungs, liver, spleen and serum using a commercially available enzyme immunoassay kit (Cayman Chemical). Isolated samples were dissociated by bead homogenization in PBS with captopril. The tissue and serum supernatants were extracted with methanol, evaporated by vacuum centrifugation, and reconstituted in a buffer provided by the Ac-SDKP EIA kit. L-PBS and L-Ac-SDKP Liposomes were diluted 1000-fold in 0.1% Triton-X and incubated overnight at 4^0C. The solution was further diluted 100-fold to 1000-fold before incubation in the Ac-SDKP EIA plate. Ac-SDKP concentration in different tissues were expressed in picograms per mg of the samples.

Induction of acute myocardial infraction:

To study the effect of L-ACSDKP on ischemic stress and cardiac function we induced acute myocardial infraction (MI) by permanent ligation of left anterior descending coronary artery as described previously[19,20]. Briefly, mice were anesthetized with ketamine (1 mg/kg intramuscular) and xylazine (5 mg/kg subcutaneous) and were intubated to undergo a ligation procedure (11-0 monofilament suture) of the left anterior descending artery. The chest wall was closed with 6-0 Vicryl sutures (Ethicon, Raritan, NJ) and Vet-bond (3M, Saint Paul, MN) followed by removal of remnant air via a 24-guage angio-needle (BD Bioscience, San Jose, CA). Ethiqa XR (3.25 mg/kg) and 1.0 mL of normal saline were injected for post procedural pain control and rehydration. The mouse was left to recover on a warm water-circulating pad (Stryker Corporation, Kalamazoo, MI) at 37°C. Acute MI was induced in 48 female mice (13-14 weeks old) of which 18 mice were used as control, 16 mice received free

Ac-SDKP via subcutaneously placed osmotic minipump and 9 mice received L-Ac-SDKP by intraperitoneal injection. Additional 5 mice were used as sham control.

Myocardial macrophage immunohistochemistry

Formalin-fixed paraffin-embedded sections (4 μm thick) were placed on charged slides and dried at 60°C for 1 hour. Slides were cooled to room temperature and added to the Dako Omnis autostainer, where they were deparaffinized and rehydrated. Sections were incubated for 30 minutes in Flex TRS high pH (Dako; catalog GV80411-2) for target retrieval. Slides were then incubated with anti-CD163 antibody (Abcam, ab182422, 1:300 dilution) for 30 minutes. Rabbit Envision (Agilent K4003, 30 minutes) followed by a Dab chromogen (5 minutes) were used for the visualization of macrophages.

Trichrome staining and evaluation of collagen content

The extent of total myocardial fibrosis was visualized by trichrome staining (Thermo Scientific Richard-Allan Scientific Masson Trichrome Kit No. 22110648). The total myocardial area and the area of positive staining for fibrosis were quantified using color deconvolution algorithms as before[7].

Supplemental tables

Table S1. Echocardiogram data

**Parameter**	**Sham (*n* = 5)**	**Control (MI) (*n* = 8)**	**L-Ac-SDKP (*n* = 7)**	**Ac-SDKP (*n* = 9)**
**Ejection fraction**	74.9 ± 5.0<br>*P* < 0.0001 *vs.* Control;<br>*P* = 0.0003 *vs.* *L-Ac-SDKP*;<br>*P* < 0.0001 *vs.* *Ac-SDKP*	48.0 ± 5.3<br>*P* = 0.0009 *vs.* *L-Ac-SDKP*;<br>*P* = 0.0246 *vs.* *Ac-SDKP*	58.5 ± 4.0<br>*P* = 0.4109 *vs.* *Ac-SDKP*	54.5 ± 3.9
**Fractional shortening**	43.4 ± 4.6<br>*P* < 0.0001 *vs.* Control;<br>*P* = 0.0001 *vs.*	24.4 ± 3.3<br>*P* = 0.0014 *vs.* *L-Ac-SDKP*;	31.0 ± 2.5<br>*P* = 0.3817 *vs.* *Ac-SDKP*	28.3 ± 2.5

	L-Ac-SDKP; *P* < 0.0001 *vs.* *Ac-SDKP*	*P* = 0.0404 *vs.* *Ac-SDKP*		
**Diameter**				
**End systolic**	2.1 ± 0.3 *P* < 0.0001 *vs.* Control; *P* = 0.0002 *vs.* *L-Ac-SDKP*; *P* < 0.0001 *vs.* *Ac-SDKP*	3.8 ± 0.6 *P* = 0.1737 *vs.* *L-Ac-SDKP*; *P* = 0.1676 *vs.* *Ac-SDKP*	3.3 ± 0.5 *P* = 0.9995 *vs.* *Ac-SDKP*	3.3 ± 0.4
**End diastolic**	3.7 ± 0.2 *P* = 0.0006 *vs.* Control; *P* = 0.0106 *vs.* *L-Ac-SDKP*; *P* = 0.0195 *vs.* *Ac-SDKP*	5.1 ± 0.7 *P* = 0.6604 *vs.* *L-Ac-SDKP*; *P* = 0.3174 *vs.* *Ac-SDKP*	4.7 ± 0.6 *P* = 0.9641 *vs.* *Ac-SDKP*	4.6 ± 0.5
**Volume**				
**End systolic**	14.6 ± 4.2 *P* < 0.0001 *vs.* Control; *P* = 0.0001 *vs.* *L-Ac-SDKP*; *P* < 0.0001 *vs.* *Ac-SDKP*	66.7 ± 24.4 *P* = 0.1591 *vs.* *L-Ac-SDKP*; *P* = 0.1559 *vs.* *Ac-SDKP*	44.9 ± 17.1 *P* = 0.9993 *vs.* *Ac-SDKP*	44.6 ± 12.5
**End diastolic**	56.9 ± 7.4 *P* = 0.0004 *vs.* Control; *P* = 0.0081 *vs.* *L-Ac-SDKP*; *P* = 0.0148 *vs.* *Ac-SDKP*	126.7 ± 39.1 *P* = 0.6047 *vs.* *L-Ac-SDKP*; *P* = 0.2878 *vs.* *Ac-SDKP*	105.5 ± 31.6 *P* = 0.9637 *vs.* *Ac-SDKP*	97.4 ± 22.9
**Stroke volume**	42.3 ± 3.8 *P* = 0.1512 *vs.* Control; *P* = 0.1268 *vs.* *L-Ac-SDKP*; *P* = 0.4924 *vs.* *Ac-SDKP*	60.0 ± 16.3 *P* = 0.9981 *vs.* *L-Ac-SDKP*; *P* = 0.7857 *vs.* *Ac-SDKP*	60.6 ± 14.6 *P* = 0.7049 *vs.* *Ac-SDKP*	52.8 ± 11.5
**LV mass**	78.7 ± 6.9 *P* < 0.0001 *vs.* Control; *P* = 0.0013 *vs.* *L-Ac-SDKP*; *P* = 0.0007 *vs.* *Ac-SDKP*	228.3 ± 62.4 *P* = 0.7446 *vs.* *L-Ac-SDKP*; *P* = 0.7459 *vs.* *Ac-SDKP*	194.9 ± 71.3 *P* = 0.9999 *vs.* *Ac-SDKP*	200.4 ± 83.7
**LV anterolateral wall**				

**End systolic**	1.3 ± 0.08<br>*P* = 0.0277 *vs.* Control;<br>*P* = 0.4073 *vs. L-Ac-SDKP*;<br>*P* = 0.6570 *vs. Ac-SDKP*	1.7 ± 0.2<br>*P* = 0.4321 *vs. L-Ac-SDKP*;<br>*P* = 0.1483 *vs. Ac-SDKP*	1.5 ± 0.2<br>*P* = 0.9449 *vs. Ac-SDKP*	1.4 ± 0.2
**End diastolic**	0.8 ± 0.005<br>*P* = 0.0089 *vs.* Control;<br>*P* = 0.1042 *vs. L-Ac-SDKP*;<br>*P* = 0.0192 *vs. Ac-SDKP*	1.0 ± 0.2<br>*P* = 0.6624 *vs. L-Ac-SDKP*;<br>*P* = 0.9663 *vs. Ac-SDKP*	0.9 ± 0.08<br>*P* = 0.8822 *vs. Ac-SDKP*	1.0 ± 0.2
**LV Posterior wall**				
**End systole**	1.0 ± 0.1<br>*P* = 0.5259 *vs.* Control;<br>*P* = 0.4086 *vs. L-Ac-SDKP*;<br>*P* = 0.4175 *vs. Ac-SDKP*	1.2 ± 0.3<br>*P* = 0.9929 *vs. L-Ac-SDKP*;<br>*P* = 0.9979 *vs. Ac-SDKP*	1.3 ± 0.3<br>*P* = 0.9996 *vs. Ac-SDKP*	1.3 ± 0.3
**End diastole**	0.6 ± 0.002<br>*P* = 0.0138 *vs.* Control;<br>*P* = 0.0141 *vs. L-Ac-SDKP*;<br>*P* = 0.0039 *vs. Ac-SDKP*	0.9 ± 0.2<br>*P* = 0.9997 *vs. L-Ac-SDKP*;<br>*P* = 0.9541 *vs. Ac-SDKP*	0.9 ± 0.2<br>*P* = 0.9769 *vs. Ac-SDKP*	1.0 ± 0.3

LV, Left ventricular.

Table S2. Primers sequences

Gene	Forward	Reverse
Acta2	AAGATCCTGACTGAGCGTGG	CCGCTGACTCCATCCCAAT
Arg1	CTCCAAGCCAAAGTCCTTAGAG	AGGAGCTGTCATTAGGGACATC
Ccl2	CCCAATGAGTAGGCTGGAGA	GCTGAAGACCTTAGGGCAGA
Cd38	ACTGGAGAGCCTACCACGAA	TGGGCCAGGTGTTTGGATTT
Col1a1	CACCCCAATCTGGTTCCCTC	CATAAGCCAAGTGGGCAGGA
Col3a1	GAGGAATGGGTGGCTATCCG	TTGCGTCCATCAAAGCCTCT
Egr2	GCGAGGAGCAAATGATGACC	GATGGGAGCGAAGCTACTCG
Lgals3	ACACGAAGCAGGACAATAACTG	TTGACCGCAACCTTGAAGTG
Il1b	TGTAATGAAAGACGGCACACC	TCTTCTTTGGGTATTGCTTGG
Il4	CTGTAGGGCTTCCAAGGTGCTTCG	CCATTTGCATGATGCTCTTTAGGC
Il6	CCGGAGAGGAGACTTCACAG	GGAAATTGGGGTAGGAAGGA
Il10	CCAAGCCTTATCGGAAATGA	TTTTCACAGGGGAGAAATCG
Mmp9	CTTCTGGCGTGTGAGTTTCCA	ACTGCACGGTTGAAGCAAAGA

Nos2	ACCTTGTTCAGCTACGCCTT	CATTCCCAAATGTGCTTGTC
Pparg	GGAAGACCACTCGCATTCCTT	TCGCACTTTGGTATTCTTGGAG
Stat6	TGAGGTGGGGACCAGCCGG	GTGACCAGGACACACAGCGG
Tgfb1	CACTCCCGTGGCTTCTAGTG	GGACTGGCGAGCCTTAGTTT
Tnfa	TCTTCTCATTCCTGCTTGTGG	GGTCTGGGCCATAGAACTGA
**18S**	CTTAGTTGGTGGAGCGATTTG	GGCTGAACGCCACTTGTCC

Acta2, actin alpha 2, smooth muscle, aorta; *Arg1*, arginase 1; *Ccl2*, monocyte chemoattractant protein-1; *Cd38*, cluster of differentiation 38; *Col1a1*, collagen type 1, alpha 1; *Col3a1*, collagen type 3, alpha 1; *Egr2*, early growth response 2; *Lgals3*, galectin-3; *Il1b*, interleukin 1 beta; *Il4*, interleukin 4; *Il6*, interleukin 6; *Il10*, interleukin 10; *Mmp9,* matrix metallopeptidase 9; *Nos2*, nitric oxide synthase 2; *Pparg*, peroxisome proliferator-activated receptor gamma; *Stat6*, signal transducer and activator of transcription 6; *Tgfb1*, transforming growth factor-beta; *Tnfa*, tumor necrosis factor alpha; 18s, 18S ribosomal RNA.

Supplemental Figures.

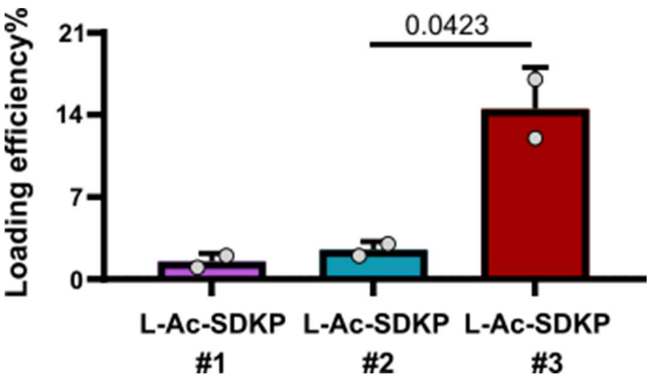

Figure S1. Optimization of liposomal formulations. Ac-SDKP encapsulation (loading) efficiency of three different liposomal formulations (#1, #2, and #3). FITC was conjugated to both free and L-Ac-SDKP. The loading efficiency was calculated according to the fluorescence intensity relative to a known concentration of Ac-SDKP.
